# Pericoronary Fat Attenuation Index and MRI-Derived Coronary Flow Reserve: A Comparative Study in Suspected Versus Known Coronary Artery Disease

**DOI:** 10.3390/tomography12040055

**Published:** 2026-04-13

**Authors:** Ryoya Takizawa, Shingo Kato, Sho Kodama, Kazuki Fukui, Ryusuke Sekii, Naofumi Yasuda, Shungo Sawamura, Tae Iwasawa, Daisuke Utsunomiya

**Affiliations:** 1 Department of Cardiology, Kanagawa Cardiovascular and Respiratory Center, Yokohama 236-0051, Japan; ryoyatakizawa@gmail.com (R.T.); sk.num10.y@gmail.com (S.K.); fukui.0o400@kanagawa-pho.jp (K.F.); 2 Department of Diagnostic Radiology, Yokohama City University Graduate School of Medicine, Yokohama 236-0004, Japan; yasuda.nao.mz@yokohama-cu.ac.jp (N.Y.); sawa0808@yokohama-cu.ac.jp (S.S.); d_utsuno@yokohama-cu.ac.jp (D.U.); 3 Department of Cardiology, Yokohama City University, Yokohama 236-0024, Japan; r.sekii0407@gmail.com; 4 Department of Diagnostic Radiology, Kanagawa Cardiovascular and Respiratory Center, Yokohama 236-0051, Japan; tae_i_md@wb3.so-net.ne.jp

**Keywords:** fat attenuation index, coronary flow reserve, computed tomography, magnetic resonance imaging, coronary artery disease

## Abstract

Background: The fat attenuation index (FAI) derived from coronary computed tomography angiography (CTA) is an emerging imaging biomarker of perivascular inflammation. Coronary flow reserve (CFR), assessed by phase-contrast (PC) cine cardiac magnetic resonance (CMR) of the coronary sinus, reflects coronary microvascular function. Although FAI has been linked to adverse outcomes in coronary artery disease (CAD), its relationship with CFR across different CAD stages is not well defined. Methods: We retrospectively evaluated 241 patients (mean age 73.4 ± 10.8 years; 149 men [61.8%]) who underwent both coronary CTA and CMR (122 with known CAD and 119 with suspected CAD). FAI was measured in the proximal left anterior descending (LAD), left circumflex (LCX), and right coronary (RCA) arteries. Impaired CFR was defined as <2.0. Univariable and multivariable logistic regression analyses were performed to identify factors associated with impaired CFR. Results: Impaired CFR was observed in 38 of 122 patients (31.1%) with known CAD and 26 of 119 (21.8%) with suspected CAD. Higher LAD-FAI was associated with impaired CFR in both groups: OR 1.06 (95% CI 1.01–1.11; *p* = 0.018) in known CAD and OR 1.08 (95% CI 1.02–1.15; *p* = 0.017) in suspected CAD. Correlation analysis also demonstrated an inverse relationship between LAD-FAI and CFR (*p* < 0.001), and the strength of association was comparable between the two groups. Conclusions: LAD-FAI was associated with impaired CFR in both suspected and known CAD, with comparable strength of association across the two groups. These findings indicate that perivascular inflammation, reflected by FAI, may relate to coronary microvascular dysfunction in different stages of CAD.

## Simple Summary

The fat attenuation index (FAI) derived from coronary computed tomography angiography reflects perivascular inflammation, whereas coronary flow reserve (CFR) assessed by phase-contrast cine cardiac magnetic resonance of the coronary sinus reflects coronary microvascular function. The relationship between FAI and CFR across different stages of coronary artery disease (CAD) remains unclear. We retrospectively evaluated 241 patients who underwent both coronary CTA and CMR, including 122 with known CAD and 119 with suspected CAD. FAI was measured in the major coronary arteries, and impaired CFR was defined as <2.0. Impaired CFR was observed in 31.1% of patients with known CAD and 21.8% with suspected CAD. Higher LAD-FAI was associated with impaired CFR in both groups and showed a significant inverse correlation with CFR. LAD-FAI was associated with impaired CFR with a similar strength of association in both suspected and known CAD, suggesting that perivascular inflammation may be linked to coronary microvascular dysfunction across different stages of CAD.

## 1. Introduction

Perivascular inflammation plays a critical role in the development and progression of coronary artery disease (CAD), contributing to endothelial dysfunction, atherosclerotic plaque formation, and adverse cardiovascular events [1,2]. The fat attenuation index (FAI), derived from coronary computed tomography angiography (CTA), has emerged as a noninvasive imaging biomarker reflecting local pericoronary inflammation [3,4,5,6]. Elevated FAI has been associated with plaque vulnerability and worse clinical outcomes in patients with known CAD, underscoring its potential role in risk stratification [7,8].

Coronary flow reserve (CFR), measured noninvasively by phase contrast cine magnetic resonance imaging (PC-CMR) of the coronary sinus, provides an integrated assessment of both epicardial and microvascular function [9,10]. Importantly, in the absence of obstructive CAD, a reduced CFR is considered indicative of coronary microvascular dysfunction, which itself has prognostic implications [11,12]. While prior studies have suggested a relationship between FAI and cardiovascular risk in patients with established CAD [13], the association between FAI and CFR—particularly in patients without obstructive CAD—remains poorly understood. Elucidating this relationship could enhance our understanding of how early perivascular inflammation contributes to microvascular impairment before the development of overt atherosclerosis.

In this study, we aimed to investigate the association between CT-derived FAI and MRI-derived CFR in patients across the spectrum of coronary artery disease (CAD). We hypothesized that, similar to patients with known CAD, those with suspected CAD would also show a significant association between elevated FAI and impaired CFR, suggesting a potential link between pericoronary inflammation and coronary microvascular dysfunction even in the absence of significant coronary artery stenosis.

## 2. Materials and Methods

### 2.1. Study Population

This retrospective observational study initially included 250 consecutive patients who underwent both coronary CT angiography and cardiac MRI. Exclusion criteria included severe valvular disease, a history of coronary artery bypass grafting, and renal dysfunction defined as a baseline serum creatinine level >1.5 mg/dL. Patients with contraindications to MRI, such as implanted cardiac devices or claustrophobia, were also excluded. For fat attenuation index (FAI) analysis, patients with left main coronary artery stenosis, chronic total occlusion, or insufficient image quality on either CT or MRI were further excluded. Among the initial cohort, 3 patients were excluded due to poor CT image quality and 6 patients due to poor MRI image quality, resulting in a final study population of 241 patients. Of these, 119 patients had no prior history of coronary artery disease (CAD) and were classified as the suspected CAD group, while the remaining 122 patients had previously diagnosed CAD and were classified as the known CAD group. All patients were referred for imaging based on clinical indications such as symptoms suggestive of myocardial ischemia, abnormal electrocardiographic findings, or post-treatment follow-up. This study was approved by the institutional review board (approval code KCRC-24-0005, 20 June 2024), and the requirement for written informed consent was waived due to its retrospective design.

### 2.2. Coronary CTA Imaging Protocol

Coronary CT imaging was performed using a 320-slice CT scanner (Aquilion ONE/GENESIS Edition, CANON medical systems, Otawara, Japan). A contrast-enhanced scan was conducted employing a standard prospectively electrocardiogram-gated protocol. The scan parameters were set as follows: tube voltage of 120 kV and tube current of 500–700 mA, which were adjusted based on the body mass index. The scanner is equipped with a 0.5 mm × 320 detector configuration. Images were reconstructed with a slice thickness of 0.5 mm and an increment of 0.3 mm using a hybrid iterative reconstruction algorithm (FC43 kernel with enhanced standard [eSTD] setting). The acquisition window and reconstruction phase were adjusted according to heart rate. For heart rates ≤ 59 bpm, images were acquired at 75% of the RR interval using single-beat acquisition. For heart rates of 60–65 bpm, single-beat acquisition at 70–80% RR was used. For heart rates of 65–70 bpm, acquisition was performed at 35–85% RR in a single beat. For heart rates of 70–80 bpm and 80–90 bpm, two-beat acquisition at 35–85% RR was used, and for heart rates ≥ 90 bpm, two- to three-beat acquisition at 35–55% RR was performed. If the heart rate exceeded 70 beats per minute during the examination, an intravenous β-blocker was administered (Lanjiolol hydrochloride, a short-acting β1-selective blocker, Ono Pharmaceutical Co., Ltd., Osaka, Japan). An iodine contrast medium (Omnipaque, GE Healthcare Pharma, Tokyo, Japan; Iopamidol, Bayer Yakuhin Ltd., Osaka, Japan), 60–70 mL, was injected into the antecubital vein at a rate of 4.5 to 5.0 mL/s. The coronary CT images were acquired using the step-and-shoot method based on the CT values of the ascending aorta.

### 2.3. FAI Measurement

**Figure 1:**
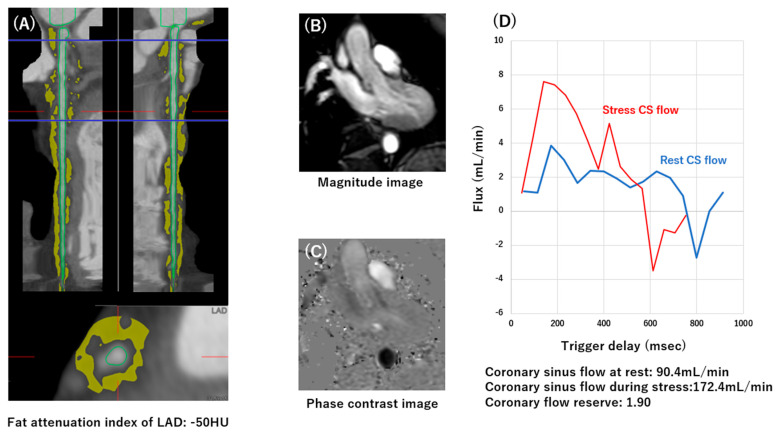
Pericoronary Adipose Tissue and Evaluation of Coronary Sinus Flow in a 70-Year-Old Female. (**A**) Curved planar reconstruction (CPR) of the left anterior descending artery (LAD) with semi-automated quantification of pericoronary adipose tissue. The fat attenuation index (FAI) of the LAD was −50 Hounsfield units (HU), which represents a markedly elevated value, indicating intense local inflammation in pericoronary fat. (**B**) Magnitude image and (**C**) phase contrast image acquired using 2D phase-contrast MRI for coronary sinus flow quantification. (**D**) Time-resolved coronary sinus flow (Flux, mL/min) plotted against trigger delay (msec) under resting (blue) and stress (red) conditions. The coronary sinus flow was 90.4 mL/min at rest and 172.4 mL/min under stress, resulting in a coronary flow reserve (CFR) of 1.90, which is severely reduced and indicates impaired coronary vasodilator function.

FAI quantification was performed using commercially available software (ZIOstation REVORAS, Ziosoft Inc., Tokyo, Japan). All measurements were conducted by an experienced cardiologist (R.T., 10 years of experience in cardiovascular medicine) who was blinded to clinical data and CFR results. FAI measurements were performed on the RCA, LAD, and LCX. The left main coronary artery was excluded from the analysis due to anatomical variability and insufficient segment length for standardized measurement. For the LAD and LCX, the proximal 40 mm of each vessel was selected for analysis. For the RCA, the segment spanning 10 to 50 mm from the ostium was used, consistent with previously validated protocols. The coronary lumen, as well as the inner and outer vessel walls, were automatically identified using dedicated software and manually adjusted when necessary to ensure accurate vessel boundary delineation. Adipose tissue surrounding the coronary artery was classified as pericoronary adipose tissue when located within a radial distance from the outer vessel wall equivalent to the vessel diameter. A voxel-based histogram of CT attenuation values within this perivascular region was generated, and the mean CT attenuation of all voxels ranging from −190 to −30 Hounsfield units was calculated. The mean attenuation value across the defined 40 mm vessel segment was used to derive the FAI (Figure 1). To assess reproducibility, interobserver and intraobserver variability were evaluated in 20 randomly selected cases by an independent cardiovascular radiologist (S.K., 20 years of experience). Interobserver and intraobserver reproducibility demonstrated excellent agreement (intraclass correlation coefficient [ICC] = 0.94 and 0.96, respectively).

### 2.4. Cardiac MRI Imaging Protocol and CFR Measurements

All examinations were performed on a 1.5 T cardiac MRI system equipped with a 32-channel coil (Ingenia Omega HP or Achieva; Philips Healthcare, Best, The Netherlands). The imaging protocol comprised ventricular function assessment, myocardial perfusion imaging under pharmacological stress and at rest, late gadolinium enhancement (LGE), and phase-contrast cine imaging (Supplementary Figure S1). Patients were positioned supine, and cardiac synchronization was achieved using vector electrocardiographic gating. After localizer scans, cine images were acquired in standard long-axis planes (two-, three-, and four-chamber views) as well as a full short-axis stack covering the left ventricle from base to apex. These images were obtained using a steady-state free precession technique with retrospective cardiac phase reconstruction. Myocardial perfusion was evaluated using a dynamic first-pass technique, in which four short-axis slices were repeatedly acquired every second heartbeat. A gadolinium-based contrast agent (gadopentetate dimeglumine or gadoterate meglumine) was administered as a bolus (0.05 mmol/kg at 4 mL/s), followed by saline. Stress imaging preceded rest imaging, with a sufficient interval (≥10 min) to allow contrast washout. For LGE assessment, an additional dose of contrast (total cumulative dose 0.2 mmol/kg) was administered after perfusion imaging. Delayed imaging was performed approximately 15 min later using an inversion-recovery gradient-echo sequence optimized to null normal myocardium. To quantify coronary sinus flow, cine images were first used to identify the vessel in the axial plane. A through-plane phase-contrast slice was then prescribed perpendicular to the coronary sinus, typically 1.5–2.0 cm distal to its ostium. Flow measurements were obtained during shallow breath-holding using a velocity-encoded gradient-echo sequence. Pharmacological stress was induced by continuous infusion of adenosine triphosphate (ATP) at 160 μg/kg/min over 4 min. Phase-contrast acquisitions were performed both during stress and under resting conditions, ensuring a recovery interval of at least 10 min between scans. To avoid attenuation of vasodilatory response, all participants were instructed to refrain from caffeine intake for at least 24 h prior to the examination.

Consistent with previous studies, coronary sinus blood flow was normalized using the rate-pressure product (RPP) as follows:

- *RPP (mmHg/min) = Systolic blood pressure (mmHg) × Heartrate (beats/min)*
- *Corrected coronary sinus flow (mL/min) = coronary sinus flow (mL/min)/RPP (mmHg/min) × 7500*

*The Δ coronary sinus flow and CFR were calculated as*

- *Δ Coronary sinus flow (mL/min) = Corrected coronary sinus flow during ATP infusion (mL/min) − Corrected coronary sinus flow at rest (mL/min)*
- *CFR = Corrected coronary sinus flow during ATP infusion (mL/min)/Corrected coronary sinus flow at rest (mL/min)*

No aliasing was observed at the selected velocity encoding (VENC) of 50 cm/s, and VENC adjustment was not required in any patient. To ensure measurement accuracy, cases with inadequate phase-contrast signal or significant motion artifacts were excluded from analysis. Interobserver and intraobserver reproducibility of coronary sinus flow and CFR measurements were assessed in 20 randomly selected cases by two independent experienced observers (R.T. and S.K.), demonstrating excellent agreement (interobserver intraclass correlation coefficient [ICC] = 0.92; intraobserver ICC = 0.96). Impaired CFR was defined as <2.0, consistent with prior coronary sinus phase-contrast CMR studies showing that this threshold is strongly associated with adverse cardiovascular outcomes [11]. Pharmacological stress was induced using intravenous adenosine triphosphate (ATP) at a dose of 160 μg/kg/min. Regadenoson is not approved for reimbursement under the Japanese national health insurance system; therefore, ATP is commonly used in clinical practice in Japan.

### 2.5. Statistical Analysis

Continuous data are expressed as either mean ± standard deviation or median with interquartile range, depending on their distribution. Categorical variables are summarized as frequencies with corresponding percentages.

Group comparisons between patients with established coronary artery disease (CAD) and those with suspected CAD were conducted using parametric or nonparametric methods as appropriate. Specifically, continuous variables were compared using Student’s *t*-test or the Mann–Whitney U test, whereas categorical variables were evaluated using the chi-square test or Fisher’s exact test. The relationship between the left anterior descending artery fat attenuation index (LAD FAI) and coronary flow reserve (CFR) was assessed using Pearson’s correlation coefficient. To explore determinants of impaired CFR (defined as CFR < 2.0), logistic regression analyses were performed. Variables demonstrating a significance level of *p* < 0.10 in univariable analysis were subsequently included in a multivariable model to identify independent predictors. Effect estimates are presented as odds ratios with corresponding 95% confidence intervals. Statistical significance was defined as a two-tailed *p* value of less than 0.05. All analyses were conducted using R (version 4.2.2; R Foundation for Statistical Computing, Vienna, Austria) and RStudio (version 2023.09.1 + 494; Posit Software, Boston, MA, USA).

Event-per-variable (EPV) was calculated for each analytical group to assess model stability, and an EPV ≥ 10 was considered adequate. Because EPV fell below this conventional threshold in subgroup analyses (Known CAD: EPV = 6.3; Suspected CAD: EPV = 4.3), results from multivariable models in these subgroups were interpreted with caution. Rather than selecting variables solely on the basis of univariable statistical significance, we constructed a pre-specified parsimonious multivariable logistic regression model based on established clinical and pathophysiological rationale. The following variables were selected a priori: age, diabetes mellitus, hypertension, LAD-FAI, LCX-FAI, and the presence of late gadolinium enhancement (LGE). RCA-FAI was not included in the primary multivariable model due to collinearity with LAD- and LCX-FAI (Pearson r > 0.7) and potential territorial mismatch with coronary sinus–derived CFR. As a sensitivity analysis, LASSO logistic regression with 10-fold cross-validation (λ selected according to the one-standard-error rule) was performed including all candidate variables. To assess internal validity and the degree of overfitting, 1000-resample bootstrap validation was conducted using the optimism method, and optimism-corrected C-statistics are reported alongside apparent C-statistics.

## 3. Results

### 3.1. Patient Characteristics

A total of 241 patients were included in the analysis, of whom 119 had suspected coronary artery disease (CAD) and 122 had known CAD. The mean age was 73.4 ± 10.8 years, and 149 patients (61.8%) were male. Patients in the known CAD group were older (74.2 ± 9.1 vs. 72.6 ± 12.3 years) and more frequently male (75.4% vs. 47.9%) compared with those in the suspected CAD group. In the suspected CAD cohort, 26 patients (21.8%) had impaired coronary flow reserve (CFR < 2.0). Compared with those with preserved CFR, patients with impaired CFR had a higher prevalence of dyslipidemia (*p* = 0.016), whereas no significant differences were observed in age, sex, or other comorbidities. In the known CAD group, 38 patients (31.1%) had impaired CFR, and these patients were more likely to have diabetes mellitus (*p* = 0.006) and dyslipidemia (*p* = 0.006) compared with those with preserved CFR, while other baseline characteristics were largely comparable (Table 1).

### 3.2. FAI in Relation to CFR, LGE, and Stress-Induced Ischemia

**Figure 2:**
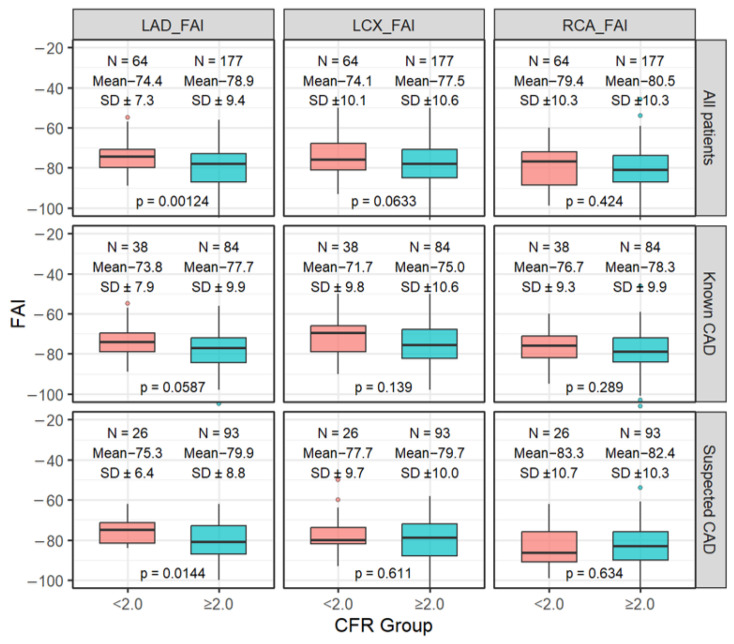
Comparison of the Coronary Fat Attenuation Index According to CFR Group in All, Known, and Suspected CAD Patients. Boxplots show the distribution of FAI in the left anterior descending (LAD), left circumflex (LCX), and right coronary arteries (RCA) stratified by CFR groups (<2.0 vs. ≥2.0). The upper, middle, and lower rows correspond to all patients, those with known CAD, and those with suspected CAD, respectively. Boxes indicate the interquartile range, horizontal lines represent the median, and dots indicate outliers. Mean ± SD values and *p*-values for between-group comparisons are displayed. A lower (more negative) FAI indicates less pericoronary inflammation.

**Figure 3:**
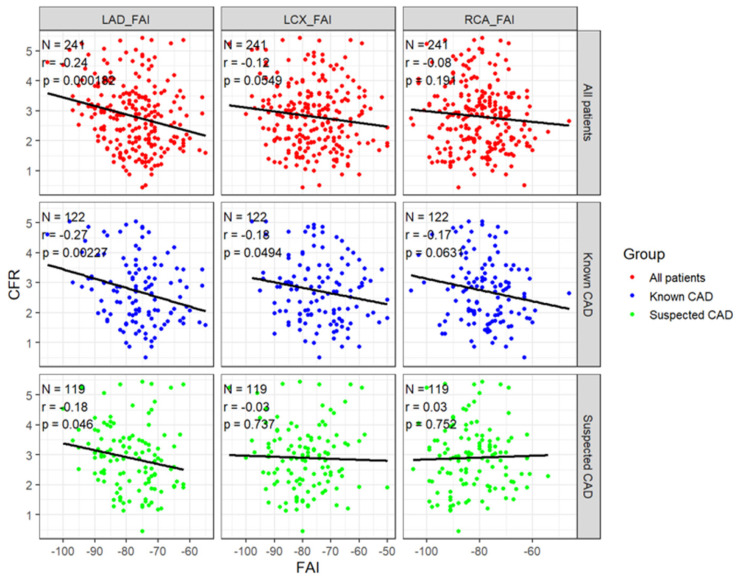
Correlation Between the Coronary Fat Attenuation Index and Coronary Flow Reserve. Scatter plots demonstrate the relationship between FAI and CFR in the left anterior descending (LAD), left circumflex (LCX), and right coronary arteries (RCA). The upper, middle, and lower rows represent all patients, patients with known CAD, and patients with suspected CAD, respectively. Each dot represents a patient, and the regression line with its slope illustrates the correlation. Pearson’s correlation coefficient (r) and corresponding *p*-value are shown in each panel. Negative correlations indicate that higher (less negative) FAI values are associated with reduced CFR, suggesting that pericoronary inflammation may relate to impaired coronary flow capacity.

When patients were stratified according to CFR status (Figure 2), LAD-FAI was higher (less negative) in those with impaired CFR compared with those with preserved CFR in the overall cohort (−74.4 ± 7.3 vs. −78.9 ± 9.4 HU; *p* = 0.0012). A similar pattern was observed in the subgroup analyses: −75.3 ± 6.4 vs. −79.9 ± 8.8 HU (*p* = 0.014) in suspected CAD and −73.8 ± 7.9 vs. −77.7 ± 9.9 HU (*p* = 0.059) in known CAD. In contrast, differences in LCX-FAI and RCA-FAI between CFR groups were less evident and did not consistently reach statistical significance. When FAI was evaluated according to the presence of myocardial tissue abnormalities on CMR, LAD-FAI did not differ significantly between patients with and without late gadolinium enhancement (LGE), whereas LCX-FAI showed a modest elevation in the LGE-positive group (Supplementary Figure S2). Analysis based on the presence of ischemia on stress imaging demonstrated a similar trend, with slightly higher LAD-FAI and LCX-FAI in the ischemia-positive group, but without significant differences (Supplementary Figure S3). Overall, LAD-FAI was most clearly associated with impaired CFR, whereas its relationship with structural myocardial abnormalities or stress-induced ischemia on CMR was less pronounced. The relationship between FAI and CFR is shown in Figure 3. LAD-FAI demonstrated an inverse correlation with CFR in the overall cohort (r = −0.24; *p* < 0.001). This association remained evident in both subgroups, with r = −0.18 (*p* = 0.046) in suspected CAD and r = −0.27 (*p* = 0.0023) in known CAD. Correlations for LCX-FAI and RCA-FAI were weaker and did not consistently reach statistical significance. These findings indicate that higher (less negative) LAD-FAI tended to accompany lower CFR, whereas the relationship for the other coronary vessels was less pronounced.

### 3.3. Logistic Regression Analysis for Predictors of Impaired CFR

Multivariable logistic regression analyses were performed to identify factors associated with impaired CFR (<2.0) in the overall cohort and in each CAD subgroup (Table 2, Table 3 and Table 4). In the overall cohort, LAD-FAI was independently associated with impaired CFR (OR 1.06; 95% CI 1.02–1.11; *p* = 0.0083), along with dyslipidemia (OR 2.33; 95% CI 1.14–5.13; *p* = 0.026) and diabetes mellitus (OR 2.11; 95% CI 1.08–4.11; *p* = 0.028). In the suspected CAD group, LAD-FAI remained an independent predictor of impaired CFR (OR 1.08; 95% CI 1.02–1.15; *p* = 0.017), together with dyslipidemia (OR 3.55; 95% CI 1.38–9.81; *p* = 0.010). Male sex showed a trend toward association (OR 2.4; 95% CI 0.94–6.50; *p* = 0.073). In the known CAD group, LAD-FAI was also associated with impaired CFR (OR 1.06; 95% CI 1.01–1.11; *p* = 0.018). Diabetes mellitus demonstrated the strongest association (OR 4.01; 95% CI 1.71–9.75; *p* = 0.0016), whereas other baseline characteristics, including age, sex, and hypertension, were not significant in the multivariable model. LCX-FAI and RCA-FAI were not independent predictors in any model.

Event-per-variable was adequate in the overall cohort (EPV = 10.7) but was below the conventional threshold of 10 in the Known CAD (EPV = 6.3) and Suspected CAD (EPV = 4.3) subgroups.

In the pre-specified multivariable model, LAD-FAI remained independently associated with impaired CFR in the overall cohort (OR 1.06, 95% CI 1.01–1.11, *p* = 0.015) and in the Suspected CAD subgroup (OR 1.09, 95% CI 1.02–1.18, *p* = 0.018). LASSO regression consistently selected LAD-FAI across all groups, whereas LCX-FAI and RCA-FAI were not retained. Bootstrap internal validation demonstrated modest optimism in the overall cohort (0.039) and Known CAD subgroup (0.050) but higher optimism in the Suspected CAD subgroup (0.086).

## 4. Discussion

To the best of our knowledge, this study is the first to evaluate the association between coronary perivascular FAI and CFR in both patients with suspected CAD and those with known CAD. The main findings can be summarized as follows. First, LAD-FAI was associated with impaired CFR (<2.0) in both suspected and known CAD. In the suspected CAD cohort (n = 119), patients with impaired CFR had significantly higher LAD-FAI compared with those with preserved CFR (−75.3 ± 6.4 vs. −79.9 ± 8.8 HU, *p* = 0.014). A similar trend was observed in the known CAD cohort (n = 122; −73.8 ± 7.9 vs. −77.7 ± 9.9 HU, *p* = 0.059), and multivariable logistic regression confirmed that LAD-FAI was an independent predictor of impaired CFR in both groups. Second, the association was largely specific to the LAD, whereas LCX-FAI and RCA-FAI showed weaker and mostly nonsignificant relationships with CFR. Third, when FAI was analyzed in relation to myocardial tissue abnormalities on CMR, only modest or inconsistent differences were observed with respect to the presence of LGE or stress-induced ischemia, although some suggestive patterns were noted, which may warrant further investigation.

FAI can be measured noninvasively from standard coronary CTA and reflects the dynamic local inflammatory milieu surrounding the coronary arteries [14,15]. Previous studies have demonstrated that higher FAI is associated with vulnerable plaque characteristics and an increased risk of major adverse cardiovascular events [3,6,7,16]. Our findings support and extend this concept by showing that elevated LAD-FAI is associated with impaired CFR even in patients without obstructive CAD, suggesting a link between perivascular inflammation and early coronary microvascular dysfunction. Notably, the strength of the association between LAD-FAI and CFR was comparable in both suspected and known CAD groups, indicating that FAI may capture early functional vascular impairment across a wide spectrum of CAD severity. This relationship’s specificity to LAD may be attributed to anatomical and physiological factors, as the LAD typically supplies a larger myocardial territory and is often affected in early atherosclerosis. In contrast, LCX-FAI and RCA-FAI demonstrated weaker associations with CFR, potentially due to smaller vessel caliber, greater susceptibility to motion artifacts, or their smaller perfusion territories. Collectively, these findings highlight the promise of LAD-FAI as a noninvasive marker of coronary microvascular dysfunction that complements functional assessment by CMR. However, its clinical utility and prognostic value require further validation in prospective studies.

CFR derived from PC-CMR has been shown to correlate well with PET, the reference standard for noninvasive coronary flow quantification, and is also reported to be a strong prognostic indicator in patients with CAD [3,4,5,6]. Our study adds to this body of evidence by linking a functional imaging biomarker (CFR) with an inflammatory imaging biomarker (FAI). Integrating these modalities may provide deeper insight into the pathophysiology of coronary vascular dysfunction. In addition, because CMR accurately detects both myocardial infarction and ischemia [17,18], we also examined the relationship between FAI and these tissue abnormalities. Although the associations were less consistent than those with CFR, several patterns emerged that may be of clinical interest. Specifically, LAD-FAI and LCX-FAI were associated with myocardial ischemia in all patients, and LCX-FAI was also associated with infarction on LGE-MRI. While the clinical interpretation of these findings remains challenging, they suggest that pericoronary inflammation, as reflected by FAI, may contribute to various forms of myocardial injury.

The combined use of FAI and CMR may contribute to optimized, individualized care by providing complementary information on distinct pathological processes. FAI reflects pericoronary inflammation, and patients with elevated FAI may benefit from anti-inflammatory therapies [19]. In contrast, abnormalities detected by CMR—such as myocardial infarction or ischemia—are typically managed with conventional CAD therapies, including antiplatelet agents and statins [20]. Therefore, integrating FAI and CMR enables simultaneous assessment of inflammation and ischemia, allowing for tailored therapeutic strategies targeting both mechanisms. However, further studies are needed to validate this approach and clarify its impact on clinical outcomes.

Beyond coronary stenosis assessment, recent advances in cardiovascular imaging increasingly emphasize substrate characterization and functional inference. In atrial arrhythmias, inflammation and tissue vulnerability have emerged as actionable substrates, as illustrated by epicardial ablation strategies in arrhythmogenic disorders and evolving ablation techniques in atrial fibrillation [21,22]. These developments underscore the clinical value of identifying pathological substrates rather than relying solely on anatomical descriptors. In parallel, the expanding role of fractional flow reserve derived from computed tomography (FFR-CT) reflects a broader shift toward machine-assisted, non-invasive functional assessment of coronary disease, including in patients with prior PCI or coronary stents [23]. Within this evolving paradigm, our integration of perivascular inflammation (FAI) and coronary microvascular function (CFR) provides a complementary framework that bridges anatomy, inflammation, and downstream physiological consequences. Future studies should determine whether such combined imaging-functional profiling improves prediction of ischemic, arrhythmic, and patient-reported outcomes across the spectrum of coronary and atrial disease. The stronger association observed for LAD-FAI may partly reflect physiological and anatomical factors. Coronary sinus flow predominantly reflects perfusion from the LAD and LCX territories, with LAD supplying the largest myocardial mass in most individuals. Therefore, inflammation within the LAD territory may have a greater impact on global coronary flow reserve derived from coronary sinus measurements. We acknowledge that coronary dominance may influence this relationship, and future studies incorporating vessel-specific perfusion assessment may further clarify this interaction.

Although EPV was adequate in the overall cohort, subgroup analyses were underpowered, particularly in the Suspected CAD group (EPV = 4.3). Accordingly, subgroup findings should be interpreted as hypothesis-generating. However, the robustness of LAD-FAI was supported by pre-specified modeling, LASSO variable selection, and bootstrap internal validation.

This study has several limitations. First, it was a retrospective, single-center analysis with a relatively small sample size, which may limit the generalizability of the findings. Second, CFR was derived from PC-CMR of the coronary sinus rather than PET, which remains the reference standard; however, prior studies have demonstrated good correlation between the two techniques. Third, clinical outcomes were not assessed, and therefore the prognostic value of FAI in relation to CFR could not be evaluated in this study. Fourth, although LAD-FAI showed a clear association with impaired CFR, the cross-sectional design precludes any inference regarding causality. Finally, measurement variability of FAI, particularly in the LCX and RCA where motion artifacts are more prominent, could have influenced the results. Finally, CT acquisition was performed using a uniform tube voltage of 120 kV to ensure attenuation stability for FAI quantification. Notably, 120 kV was also used in the majority of cases in the large-scale landmark FAI study [11]. Nevertheless, because FAI values are influenced by tube voltage and reconstruction parameters, absolute FAI values may not be directly generalizable to protocols using lower or automated kV settings.

## 5. Conclusions

LAD-FAI was associated with impaired CFR in both suspected and known CAD cases, with comparable strength of association across the two groups. This relationship was predominantly LAD-specific, whereas LCX-FAI and RCA-FAI showed weaker correlations. These results indicate that perivascular inflammation, reflected by FAI, may be linked to coronary microvascular dysfunction across the spectrum of CAD. The integration of CTA-derived FAI with CMR-derived CFR may provide complementary insights into coronary pathophysiology, although further prospective studies are required to establish its prognostic and clinical significance.

## Figures and Tables

**Table 1:** Patients’ Characteristics.

Variable	All Patients (n = 241)	Known CAD (n = 122)	Suspected CAD (n = 119)	*p*-Value
**Age**	73.4 ± 10.8	74.2 ± 9.1	72.6 ± 12.3	0.72
**BMI**	24.0 ± 4.0	23.9 ± 3.7	24.1 ± 4.3	0.96
**Male**	149 (61.8%)	92 (75.4%)	57 (47.9%)	<0.001
**Symptom**	88 (36.5%)	65 (53.3%)	23 (19.3%)	<0.001
**Smoker**	33 (13.7%)	20 (16.4%)	13 (10.9%)	0.26
**HT**	183 (75.9%)	99 (81.1%)	84 (70.6%)	0.070
**Dyslipidemia**	166 (68.9%)	112 (91.8%)	54 (45.4%)	<0.001
**DM**	56 (23.2%)	39 (32.0%)	17 (14.3%)	0.006
**LBBB**	28 (11.6%)	15 (12.3%)	13 (10.9%)	0.84
**Resting ST segment changes**	24 (10.0%)	14 (11.5%)	10 (8.4%)	0.52
**Q wave**	41 (17.0%)	23 (18.5%)	18 (15.5%)	0.60
**Negative T wave**	24 (10.0%)	11 (9.0%)	13 (10.9%)	0.67
**Poor R progression**	4 (1.7%)	4 (3.3%)	0 (0.0%)	0.12
**Aspirin**	132 (54.8%)	115 (94.3%)	17 (14.3%)	<0.001
**Statin**	166 (68.9%)	152 (91.8%)	14 (11.8%)	<0.001
**ACI/ARB**	121 (50.2%)	59 (48.4%)	62 (52.1%)	0.60
**Beta**	84 (34.9%)	54 (44.3%)	30 (25.2%)	0.003
**Ca blocker**	88 (36.5%)	52 (42.6%)	36 (30.3%)	0.061
**SGLT2 inhibitor**	29 (17.4%)	20 (16.4%)	10 (8.4%)	0.049
**Diabetic drugs**	29 (12.0%)	20 (16.4%)	9 (7.6%)	0.047
**LAD_FAI**	−77.7 ± 9.1	−76.5 ± 9.5	−78.9 ± 8.6	0.061
**LCX_FAI**	−76.6 ± 10.5	−73.8 ± 9.4	−79.3 ± 10.0	<0.001
**RCA_FAI**	−80.2 ± 10.3	−77.8 ± 9.7	−82.6 ± 10.3	<0.001
**LVEDV**	123.5 ± 44.8	124.6 ± 46.4	122.3 ± 43.4	0.70
**LVEF**	58.7 ± 12.5	57.9 ± 13.3	59.4 ± 11.8	0.54
**LVESV**	54.7 ± 38.5	56.4 ± 40.3	52.9 ± 36.1	0.59
**LV mass**	74.1 ± 28.4	75.5 ± 25.6	72.8 ± 31.1	0.15
**LGE presence**	51 (21.2%)	42 (34.4%)	9 (7.6%)	<0.001
**Ischemia presence**	34 (14.1%)	25 (20.5%)	9 (7.6%)	0.005
**CFR**	2.8 ± 1.1	2.7 ± 1.1	2.9 ± 1.1	0.17

Values are presented as mean ± SD or n (%). *p*-values indicate comparisons between patients with known CAD and suspected CAD. ACEI/ARB = angiotensin-converting enzyme inhibitor/angiotensin II receptor blocker; BMI = body mass index; Ca blocker = calcium-channel blocker; CFR = coronary flow reserve; DM = diabetes mellitus; HT = hypertension; LAD_FAI = fat attenuation index of the left anterior descending coronary artery; LBBB = left bundle branch block; LCX_FAI = fat attenuation index of the left circumflex coronary artery; LGE = late gadolinium enhancement; LV mass = left ventricular mass; LVEDV = left ventricular end-diastolic volume; LVEF = left ventricular ejection fraction; LVESV = left ventricular end-systolic volume; RCA_FAI = fat attenuation index of the right coronary artery; SGLT2 inhibitor = sodium-glucose cotransporter-2 inhibitor.

**Table 2:** Univariate and Multivariate Logistic Regression Analysis for Predicting CFR < 2.0.

	Univariable Logistic Regression Analysis			Multivariable Logistic Regression Analysis		
Variable	OR	95% CI	*p*-Value	OR	95% CI	*p*-Value
**A** **ge**	0.99	0.96–1.02	0.43	-	-	-
**male**	1.84	1.0–3.48	0.055	-	-	-
**BMI**	1.0	0.93–1.07	0.97	-	-	-
**smoker**	1.24	0.53–2.71	0.6	-	-	-
**HT**	1.34	0.68–2.77	0.41	-	-	-
**Dyslipidemia**	2.73	1.37–5.84	0.0061	2.33	1.14–5.13	0.026
**DM**	2.45	1.29–4.62	0.0057	2.11	1.08–4.11	0.028
**LAD_FAI**	1.06	1.02–1.1	0.0010	1.06	1.02–1.11	0.0083
**LCX_FAI**	1.03	1.0–1.06	0.029	0.99	0.96–1.03	0.76
**RCA_FAI**	1.01	0.98–1.04	0.45	-	-	-
**LGE presence**	0.61	0.27–1.27	0.20	-	-	-
**Ischemia presence**	1.18	0.51–2.57	0.68	-	-	-
**LVEDV**	1.01	1.0–1.01	0.10	-	-	-
**LVEF**	0.99	0.97–1.01	0.22	-	-	-
**LVESV**	1.01	1.0–1.01	0.077	-	-	-
**LV mass**	1.01	1.0–1.02	0.16	-	-	-

BMI = body mass index; CFR = coronary flow reserve; CI = confidence interval; DM = diabetes mellitus; FAI = fat attenuation index; HT = hypertension; LAD = left anterior descending coronary artery; LCX = left circumflex coronary artery; LGE = late gadolinium enhancement; LV = left ventricle; LVEDV = left ventricular end-diastolic volume; LVEF = left ventricular ejection fraction; LVESV = left ventricular end-systolic volume; OR = odds ratio; RCA = right coronary artery.

**Table 3:** Univariate and Multivariate Logistic Regression Analyses for Predicting CFR < 2.0 in Known CAD.

	Univariable Logistic Regression Analysis			Multivariable Logistic Regression Analysis		
Variable	OR	95% CI	*p*-Value	OR	95% CI	*p*-Value
**age**	0.99	0.95–1.03	0.57	-	-	-
**male**	1.07	0.45–2.73	0.87	-	-	-
**BMI**	1.00	0.90–1.11	0.98	-	-	-
**smoker**	1.23	0.43–3.32	0.68	-	-	-
**HT**	1.80	0.65–5.83	0.28	-	-	-
**Dyslipidemia**	1.89	0.45–12.98	0.43	-	-	-
**DM**	3.80	1.69–8.73	0.0013	4.01	1.71–9.75	0.0016
**LAD_FAI**	1.05	1.00–1.10	0.038	1.06	1.01–1.11	0.018
**LCX_FAI**	1.03	0.99–1.07	0.10	-	-	-
**RCA_FAI**	1.02	0.98–1.06	0.39	-	-	-
**LGE presence**	0.39	0.15–0.93	0.040	0.41	0.15–1.03	0.066
**Ischemia presence**	0.83	0.30–2.12	0.70	-	-	-
**LVEDV**	1.01	1.00–1.01	0.21	-	-	-
**LVEF**	1.00	0.97–1.02	0.73	-	-	-
**LVESV**	1.00	1.00–1.01	0.28	-	-	-
**LV mass**	1.01	0.99–1.02	0.26	-	-	-

BMI = body mass index; CAD = coronary artery disease; CFR = coronary flow reserve; CI = confidence interval; DM = diabetes mellitus; FAI = fat attenuation index; HT = hypertension; LAD = left anterior descending coronary artery; LCX = left circumflex coronary artery; LGE = late gadolinium enhancement; LV = left ventricle; LVEDV = left ventricular end-diastolic volume; LVEF = left ventricular ejection fraction; LVESV = left ventricular end-systolic volume; OR = odds ratio; RCA = right coronary artery.

**Table 4:** Univariate and Multivariate Logistic Regression Analyses for Predicting CFR < 2.0 in Suspected CAD.

	Univariable Logistic Regression Analysis			Multivariable Logistic Regression Analysis		
Variable	OR	95% CI	*p*-Value	OR	95% CI	*p*-Value
**age**	0.99	0.95–1.02	0.46	-	-	-
**male**	2.5	1.03–6.43	0.0473	2.4	0.94–6.5	0.073
**BMI**	1	0.9–1.11	0.927	-	-	-
**smoker**	1.08	0.23–3.89	0.91	-	-	-
**HT**	0.92	0.37–2.47	0.864	-	-	-
**Dyslipidemia**	2.86	1.18–7.36	0.0234	3.55	1.38–9.81	0.010
**DM**	0.74	0.16–2.5	0.652	-	-	-
**LAD_FAI**	1.07	1.01–1.13	0.0174	1.08	1.02–1.15	0.017
**LCX_FAI**	1.02	0.98–1.07	0.349	-	-	-
**RCA_FAI**	0.99	0.95–1.03	0.713	-	-	-
**LGE presence**	1.02	0.15–4.57	0.978	-	-	-
**Ischemia presence**	1.89	0.38–7.76	0.392	-	-	-
**LVEDV**	1	1–1.01	0.31	-	-	-
**LVEF**	0.98	0.94–1.01	0.178	-	-	-
**LVESV**	1.01	1–1.02	0.166	-	-	-
**LV mass**	1.01	0.99–1.02	0.426	-	-	-

BMI = body mass index; CAD = coronary artery disease; CFR = coronary flow reserve; CI = confidence interval; DM = diabetes mellitus; FAI = fat attenuation index; HT = hypertension; LAD = left anterior descending coronary artery; LCX = left circumflex coronary artery; LGE = late gadolinium enhancement; LV = left ventricle; LVEDV = left ventricular end-diastolic volume; LVEF = left ventricular ejection fraction; LVESV = left ventricular end-systolic volume; OR = odds ratio; RCA = right coronary artery.

## Data Availability

The restriction is due to ethical restrictions, as the datasets contain sensitive patient information and are subject to approval by the institutional ethics committee.

## Supplementary Materials

The following supporting information can be downloaded at: https://www.mdpi.com/article/10.3390/tomography12040055/s1, Supplemental Figure S1. Study Protocol of Cardiac MRI; Supplemental Figure S2. Coronary Fat Attenuation Index According to the Presence of Late Gadolinium Enhancement; Supplemental Figure S3. Coronary Fat Attenuation Index According to the Presence of Myocardial Ischemia.
