# Supplementary material for: Pericoronary Fat Attenuation Index and MRI-Derived Coronary Flow Reserve: A Comparative Study in Suspected Versus Known Coronary Artery Disease
Source: Tomography. 2026 Apr 13;12(4):55. doi: 10.3390/tomography12040055 (PMC13119742; doi:10.3390/tomography12040055)
Supplement: Supplementary file 1 [file tomography-12-00055-s001.zip › tomography-4149513-SI.pdf]

Supplemental Figure

Table S1. EPV and Bootstrap Validation

Study Group	N	Events (CFR<2.0)	EPV	Apparent C	Corrected C (Bootstrap)	Optimism
All patients	241	64	10.7 ✓	0.680	0.641	0.039
Known CAD	122	38	6.3 ⚠	0.746	0.696	0.050
Suspected CAD	119	26	4.3 ⚠	0.687	0.602	0.086

Table S2. LASSO-Selected Variables (λ.

Study Group	LASSO-selected variables (λ.1se)
All patients	HLA (β=0.1266)<br>DM (β=0.1191)<br>LAD_FAI (β=0.0164)
Known CAD	DM (β=0.4960)<br>LAD_FAI (β=0.0023)
Suspected CAD	male (β=0.1068)<br>HLA (β=0.3138)<br>LAD_FAI (β=0.0205)

Figure S1. Forest Plot — Univariable and Multivariable Logistic Regression

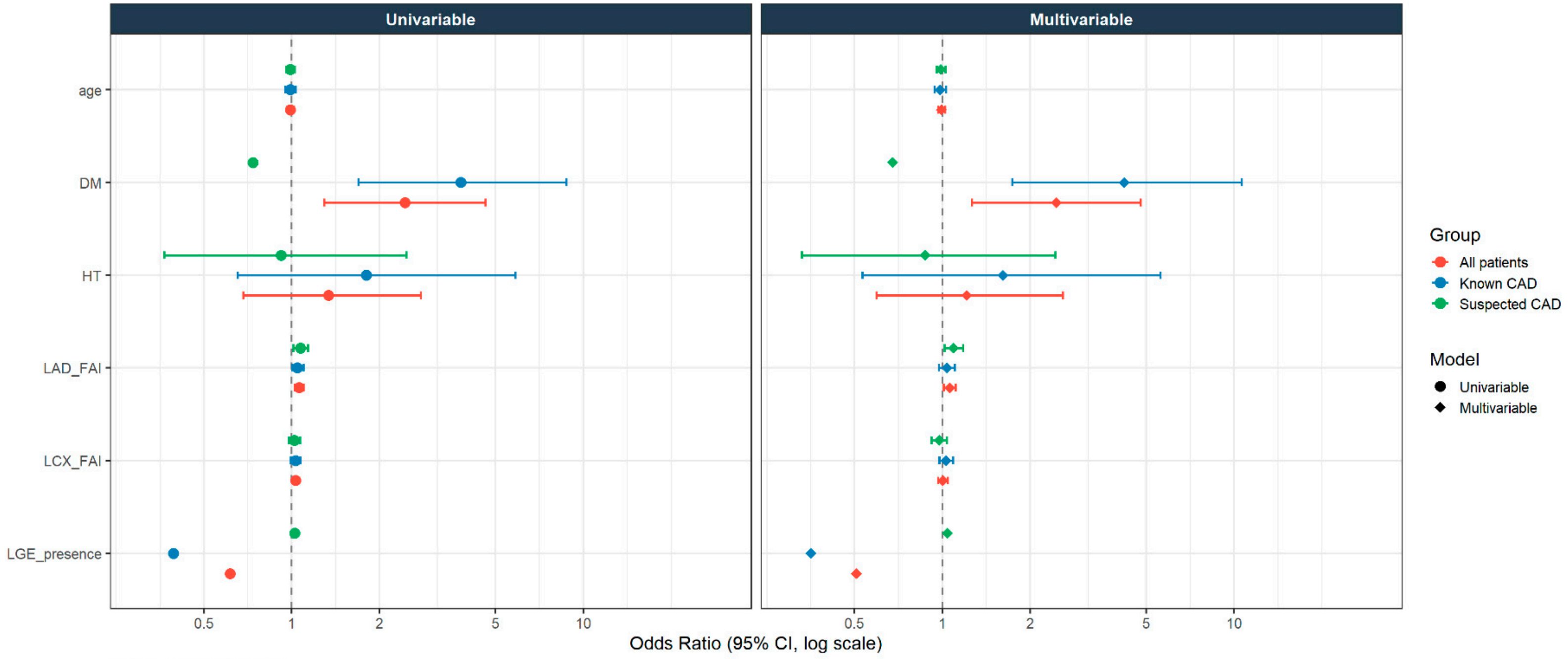

* p<0.05 ** p<0.01 *** p<0.001
Events: CFR < 2.0 (impaired coronary flow reserve)

Event definition: CFR < 2.0 (impaired coronary flow reserve). Prespecified variables: age, DM, HT, LAD_FAI, LCX_FAI, LGE_presence.
EPV = Events Per Variable. Bootstrap B = 1000 resamples (Harrell optimism method). LASSO: 10-fold CV, alpha=1, lambda.1se. CFR >5.5
